# MWCNT/rGO/natural rubber latex dispersions for innovative, piezo-resistive and cement-based composite sensors

**DOI:** 10.1038/s41598-021-98596-3

**Published:** 2021-09-23

**Authors:** L. Verdolotti, C. Santillo, G. Rollo, G. Romanelli, M. Lavorgna, B. Liguori, G. C. Lama, E. Preziosi, R. Senesi, C. Andreani, M. di Prisco

**Affiliations:** 1 Institute of Polymers, Composite and Biomaterials, National Research Council, , P.Le Enrico Fermi, 1, 80055 Portici, Naples, Italy; 2 Institute of Polymers, Composite and Biomaterials, National Research Council, Via Previati 1/C, 23900 Lecco, Milan, Italy; 3 Rutherford Appleton Laboratory, ISIS Facility, Chilton, Didcot, Oxfordshire, OX11OQX UK; 4 Department of Chemical, Materials and Production Engineering, University of Naples Federico II, P.le Tecchio, 1, 80138 Naples, Italy; 5 Dipartimento di Fisica and NAST Centre, Università degli Studi di Roma “Tor Vergata”, Via della Ricerca Scientifica 1, 00133 Rome, Italy; 6 Department of Civil and Environmental Engineering, Politecnico di Milano, P.za Leonardo da Vinci, 32, 20133 Milan, Italy

**Keywords:** Engineering, Materials science, Nanoscience and technology

## Abstract

The present study is focused on the development and characterization of innovative cementitious-based composite sensors. In particular, multifunctional cement mortars with enhanced piezoresistive properties are realized by exploiting the concept of confinement of Multiwall Carbon Nanotubes (MWCNTs) and reduced Graphene Oxide (rGO) in a three-dimensional percolated network through the use of a natural-rubber latex aqueous dispersion. The manufactured cement-based composites were characterized by means of Inelastic Neutron Scattering to assess the hydration reactions and the interactions between natural rubber and the hydrated-cement phases and by Scanning Electron Microscopy and X-Ray diffraction to evaluate the morphological and mineralogical structure, respectively. Piezo-resistive properties to assess electro-mechanical behavior in strain condition are also measured. The results show that the presence of natural rubber latex allows to obtain a three-dimensional rGO/MWCNTs segregate structure which catalyzes the formation of hydrated phases of the cement and increases the piezo-resistive sensitivity of mortar composites, representing a reliable approach in developing innovative mortar-based piezoresistive strain sensors.

## Introduction

In the civil-construction sector, the new global technological standards, requiring increasingly “smart” infrastructures, are guiding the development of building materials with multifunctional characteristics. In particular, research, development and innovativeness are paving the way towards the creation of innovative multifunctional construction materials (mainly mortars and concretes) with improved mechanical properties as well as functional properties such as thermal insulation, electrical conductivity, self-cleaning, photo-catalysis, etc., through the tailoring of the nanometer structure of the cementitious phase. In fact, since most of the mortar and concrete damages occur in cement-based binding materials, it is possible to improve them by tracing back to their chemical and mechanical defects, designing more effective micron and sub-micron structures by exploiting the potentials of nanoparticles^1,2^.

In this respect, the development of cement-based composites by using carbonaceous fillers (i.e. Multiwall Carbon Nanotubes (MWCNTs), Graphene and/or its derivatives) able to strengthen the structure of cement hydrated phases, and enabling the achievement of additional functionalities including the piezo-resistive properties useful for realizing strain sensors for the structural health monitoring of buildings, is a very topical issue, still to be fully explored^3,4^. The enhancement of structural and functional properties of cement-based composites realizes when the nanometric filler is loaded at a defined concentration, i.e. the percolation threshold, which enables the formation of a continuous three-dimensional network throughout the cementitious matrix. The filler amount to get the percolation depends on the geometrical properties of the filler (i.e. shape, 1- or 2-dimensional nature of the filler, and aspect ratio) and, generally, the best improvements of structural and functional performances are observed for filler contents from 1 wt% (with respect to the cement fraction) up to several percentage units^5–8^. Moreover, the peculiarities of cementitious-based composites strongly depend on the ability to homogeneously distribute the carbonaceous filler within the mortar and concrete volume, without destroying the integrity of the cement-based hydrated phase and avoiding any marked filler aggregation and/or separation. Furthermore, to obtain an improvement in the mechanical and functional properties of the resulting composites, it is required a good interaction at the carbonaceous filler-matrix interface. Generally, the strong tendency of these fillers to agglomerate due to the presence of attractive forces (e.g., Van der Waals), can hinder the positive effect of the filler. Thus, in order to control the distribution homogeneity of the carbonaceous fillers (carbon nanotubes (CNTs), graphene and its derivatives) into organic or inorganic matrices, various methods have been proposed including physical techniques, such as ultrasonication, ball milling and mechanical stirring, or chemical methods, such as the use of surfactants, covalent functionalization of fillers or a combination of these methods^4,9–13^. As far as the composites with inorganic matrix are concerned, all these methods promote the distribution of the carbonaceous filler in the whole binding phase generated from the hydration of the cement which, being a massive phase, requires a huge filler content to get an effective three-dimensional percolation, thus resulting in the costs/performance balance as a material with either poor electrical properties or highly expensive.

In this context, several approaches have been carried out on the use of carbonaceous fillers in order to promote the cement hydration as well as to improve the resulting structural, morphological and functional properties of cementitious-based systems^14^. In particular, Bai et al.^15^ showed that the introduction of silica fume, facilitating the graphene dispersion and increasing the interfacial strength between the filler and cement matrix, enhances the compressive strength and electrical properties of the resulting composites. Zhan et al.^7^ synthesized CNTs directly on the surface of fly ash (CNT-coated FA) and then these particles were incorporated into cement mortars. The composite with a 2 wt% of CNT-coated FA exhibited good mechanical and piezoresistive properties. Some research also used a mixture of carbonaceous filler for the development of mortar-composites. For example, Han et al.^8^ added both CNTs and carbon black into cement mortars enhancing their electrical conductivity and endowing them of stable and sensitive piezoresistivity.

Natural rubber (NR) latex obtained from the Hevea brasiliensis tree is an environmentally friendly and low-cost material, which can be easily processed with well-established technologies^16^. These sustainable peculiarities, combined with its excellent mechanical properties, make the natural rubber an ideal candidate to realize composites useful for many applications, e.g., tires, seals, shock absorptions^16^, flexible strain sensors^16,17^ and as biomaterials for tissue repair^18^.

Recently, some of the authors have developed an innovative approach to prepare natural rubber-based composite materials containing the carbonaceous filler (i.e. carbon nanotubes and graphene derivatives) in a well-tailored three-dimensional morphology. This method involves the dispersion of the filler in an aqueous-rubber latex dispersion and its assembling on the surface of the latex particles. Thus it is possible to both avoiding filler aggregation and realizing a three-dimensional network with the fillers localized only in between the interstices of the single latex particles which randomly coalesce to produce a continuous rubbery phase^19^. This approach is very effective and allows to reducing the amount of filler needed to reach a percolating-network into the polymeric matrix (as compared to the amount needed for achieving the geometrical percolation)^20^. This concept can be translated to the cement-based composite materials, in order to promote the segregation of the conductive filler wherein the rubber latex particles coalesce, thus allowing the three-dimensional filler percolation with a filler amount lower than that needed when the filler is randomly distributed throughout the cement-based hydrated phase.

Despite the huge efforts addressed to investigate the realization of cementitious composites with carbonaceous fillers, there are only a few scientific papers focused on the understanding of peculiar role of the carbonaceous fillers in the chemical hydration processes of cement and formation of new hydrated phases (namely the micro and nanostructure of calcium silicon hydrates, CSH) and in the mechanisms of mechanical improvement and exploitation of functional properties^21,22^.

Insights about the physical and chemical properties of hydrated cement can be achieved by means of Inelastic Neutron Scattering (INS)^23,24^. INS is an experimental technique probing lattice, inter- and intramolecular vibrations in condensed matter systems^25^, similarly to infra-red and Raman spectroscopies^26–28^, yet without selection rules. Experimental spectra display a dominant scattering contribution from hydrogen, in the so-called incoherent approximation^29^, making INS an exquisite technique to capture lattice modes in molecular systems, such as water and latex. As neutron scattering is particularly sensitive to hydrogen, as opposed to photon-based techniques where the signal is proportional to the atomic number, INS provides an important complementary information to vibrational techniques such as Raman and infra-red spectroscopies (see e.g.,^30^). Moreover, owing to the high penetration depth achieved in neutron experiments, INS can be applied to real-size and bulk materials, still providing a description of the system at the atomic scale. Over the last decades, INS investigations have tackled the hydration mechanism in traditional cements by probing the creation of Ca-OH bonds^31^, and the hydration mechanism and water dynamics in concrete^32^ and isolated cement components^33,34^. However, investigations through INS on cementitious composites with carbonaceous fillers are still missing.

Starting from this background, cement-based composites modified with carbonaceous fillers previously assembled on rubber latex particles, were prepared through the following multi-steps approach:

- Selection of the suitable carbonaceous fillers (Multiwall Carbon Nanotubes-MWCNT, graphene derivatives and a mixture of them) and the dispersion media (rubber-based latex dispersion);
- Mix design of the cement-based composite formulation by selecting the amount of filler and other components, such as cement, water, and additives;
- Assembling of reduced graphene oxide and MWCNT particles onto the latex particles by ultrasonication mixing, optimization of workability of the resulting cement-based mixture and composites casting molding and hydration curing.

The produced cement-based composites (namely mortar composites MC) containing the carbonaceous fillers, were characterized by means of INS to evaluate the chemical hydration of anhydrous cementitious phases, Scanning Electron Microscopy (SEM) and X-Ray diffraction to evaluate, respectively, the morphological and mineralogical structure and piezo-resistive properties to assess their electro-mechanical properties in strain condition.

## Results and discussion

### Chemical characterization by Inelastic Neutron Scattering

**Figure 1:**
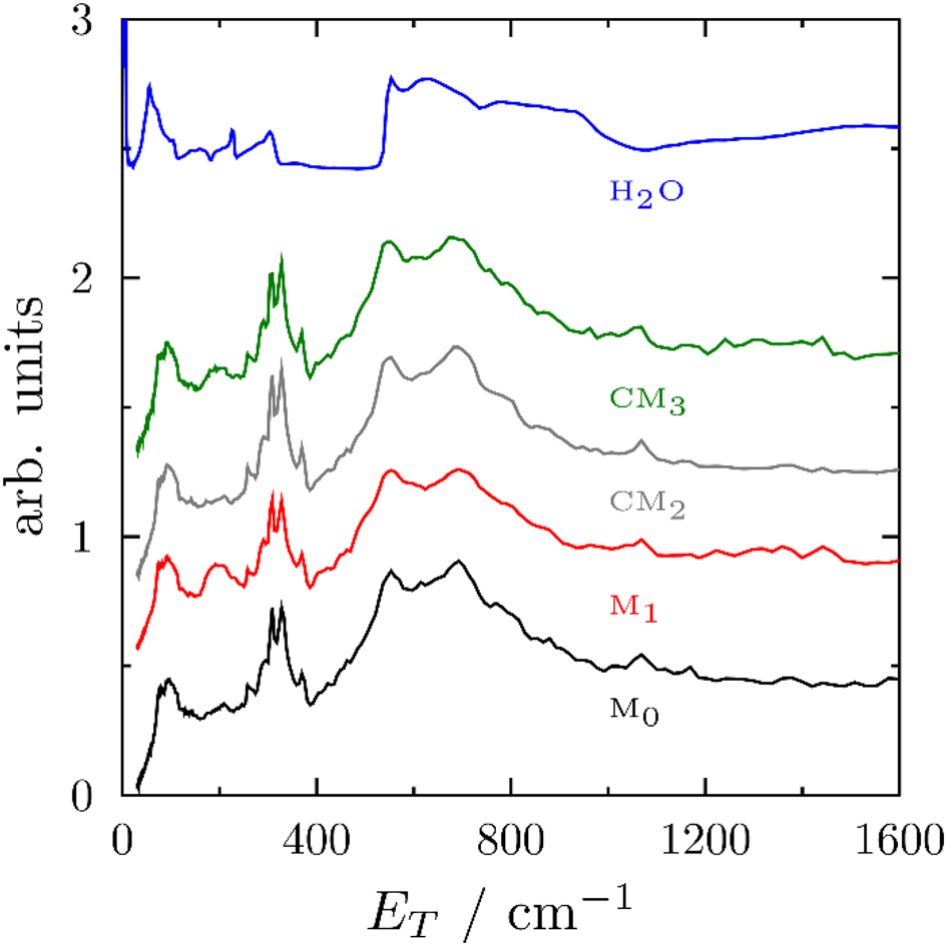
Experimental INS spectra of M_0_ (black); M_1_ (red); CM_2_ (grey); CM_3_ (green); and bulk H_2_O (blue). Spectra have been scaled and shifted vertically to ease visualization and comparison.

Figure 1 shows the INS spectra of M_0_, M_1_, CM_2_ and CM_3_ mortar samples after subtraction of the backgrounds from dry cement and empty container. As the main contribution to the spectra comes from hydrogen atoms involved in cement hydration, data were compared with a deionized H_2_O sample measured at the same temperature, also shown in Fig. 1. From the bulk-H_2_O spectrum, it is possible to recognize the translational (below 400 cm^−1^) and vibrational (around 600 cm^−1^) modes arising from the intermolecular interactions and the hydrogen-bonding network. By comparison, it is noticed how the sharp vibrational feature in bulk H_2_O is clearly broadened and red-shifted in the mortar samples, as a consequence of the different interactions that coordinated water molecules experience within the mortar structure. The overall shift of the vibrational bands to lower frequencies corresponds to a picture whereby some H_2_O molecules undergo rotations that are less hindered than in the bulk, due to the breaking of the hydrogen-bonding network. At lower energies, the sharp translational features in bulk water at 50 cm^−1^, 220 cm^−1^, and 305 cm^−1^ disappear, replaced by more complex lattice and translational motions between 70 and 400 cm^−1^ in the hydrated cements, resulting from the coordination of water to the cement, as well as the creation of new CSH species. In particular, the strong peak at ca. 330 cm^−1^ corresponds to the Ca–OH bond^31^. Molecular modelling^35^ and far-IR spectra^36^ of CSH phases also showed peaks in the same region, related to the vibrations of Ca(OH)_2_ grains forming in the mesopores of the cementitious material. Apart from minor differences related to NR, discussed below, it is worth noting how the spectra of the four mortar samples, with and without fillers and NR, closely resemble each other. This brings to the conclusion that the average structure and dynamics of hydrogen within the mortars is not affected by the inclusion of rGO, MWCNT, and NR at the investigated concentrations. The same conclusion can be drawn from the spectra of filler dispersions that well reproduced that of bulk H_2_O (the spectra are not shown for sake of brevity).

**Figure 2:**
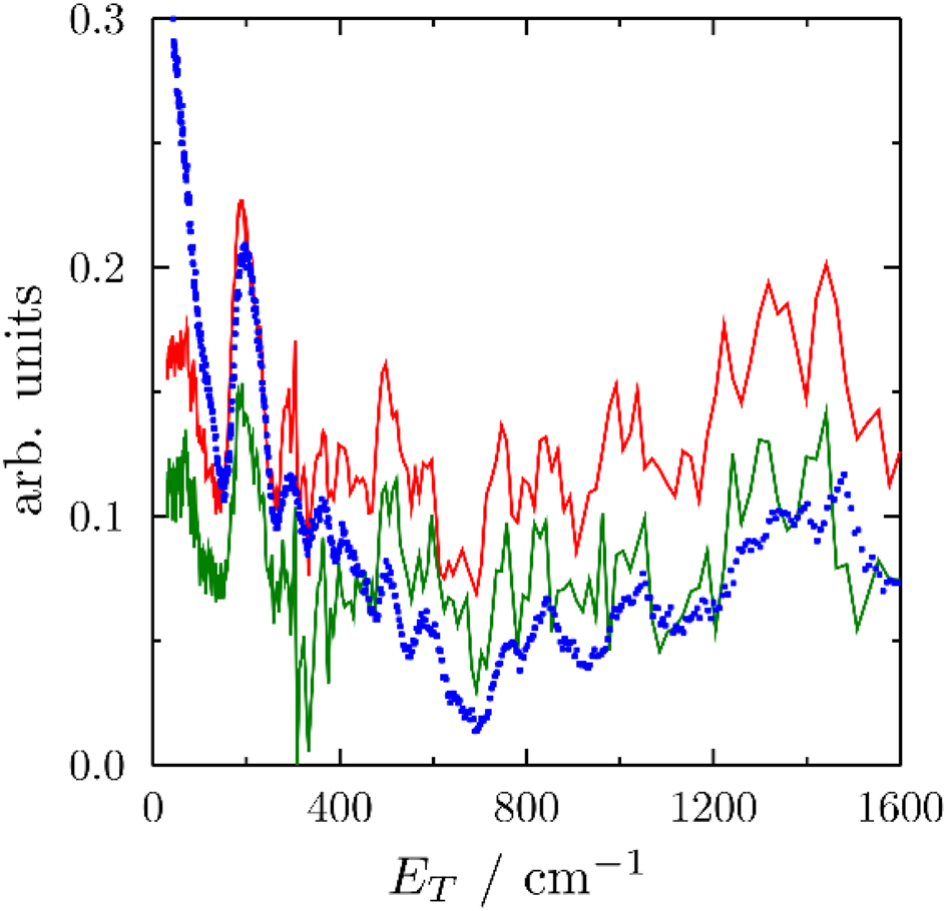
Experimental INS spectra of natural rubber from M_1_ (red); CM_2_ (green); and its bulk phase (blue) from Ref.^37,38^.

The two mortar systems including the NR dispersions, of which the main component is polyisoprene (PIP), manifest an additional intensity clearly visible around 200 cm^−1^ in Fig. 1, likely related to the CH_3_ torsion mode in PIP^37^. The signal from PIP in the two composites was isolated by subtraction of the corresponding sample prepared without natural rubber. The results are shown in Fig. 2 and compared to the spectra from Adams et al.^37^ available from the TOSCA INS Database^38^. Despite some noise in the resulting spectra, related to the small amount of natural rubber in the sample (about 1 wt%), the features from PIP in both mortar samples, with and without carbonaceous fillers, closely resemble those from bulk PIP. A slight shift in the first peak (200 cm^−1^) is likely related to the fact that the reference spectrum was measured on a previous version of the TOSCA spectrometer (TFXA), with lower resolution at the elastic line (0 cm^−1^) providing a larger background below 200 cm^−1^. Because of such background, it is difficult to infer if the clear feature at around 100 cm^−1^ was absent in bulk PIP. However, over the entire range of intermolecular vibrations, the peak positions in both mortar samples closely match those from Adams et al.^37^. Such similarities in the region of intermolecular vibrations highlight that negligible interactions take place between NR and the rest of cementitious-based hydration phases and other components of the mortar matrix.

### Structural and morphological characterization

**Figure 3:**
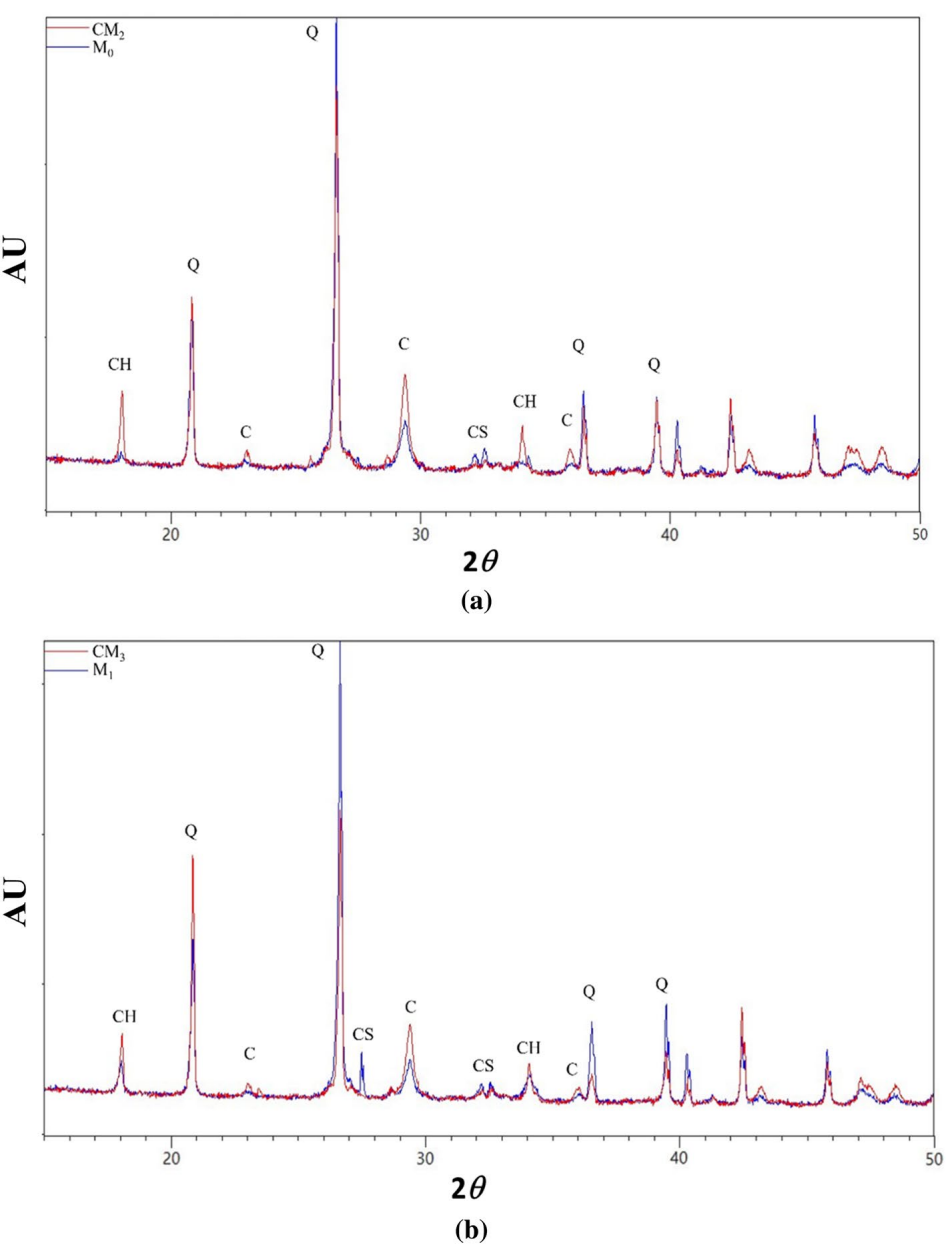
X-ray diffraction spectra of mortar samples after the hydration process (CH is for calcium hydroxide, C for calcium carbonate, CS for calcium silicate and Q is for quartz mineral phases).

The effect of carbonaceous fillers on the hydration of cementitious phases can be investigated by analyzing the crystalline phases present in each mortar sample at the end of the hydration curing process. M_0_ and M_1_ samples (blue line in Fig. 3a, b) show quartz as main crystalline phase (silica sand, ICCD #01-083-2465) and present also traces of un-hydrated calcium silicate (C_3_S, ICCD #01-086-0402) (see the most intense reflection at 2*θ* equal to 26.63° and 29.40° respectively). Otherwise, CM_2_ and CM_3_ composites (red line in Fig. 3a, b) showed traces of typical crystalline hydrated products such as calcium hydroxide, CH (ICCD # ICCD # 00-033-0306, the main peak is at 2*θ* equal to 36.55°) for CM_3_ (diffraction peak at 35°) were clearly detected^39,40^. These diffraction features confirm that MWCNTs and rGO carbonaceous fillers are able to speed-up the hydration kinetics of the cementitious phases in the mortar, as already verified by Lin et al.^41^, enhancing the assembly of the CH crystals produced during the hydration (mainly for the CM_3_) process^42^.

**Figure 4:**
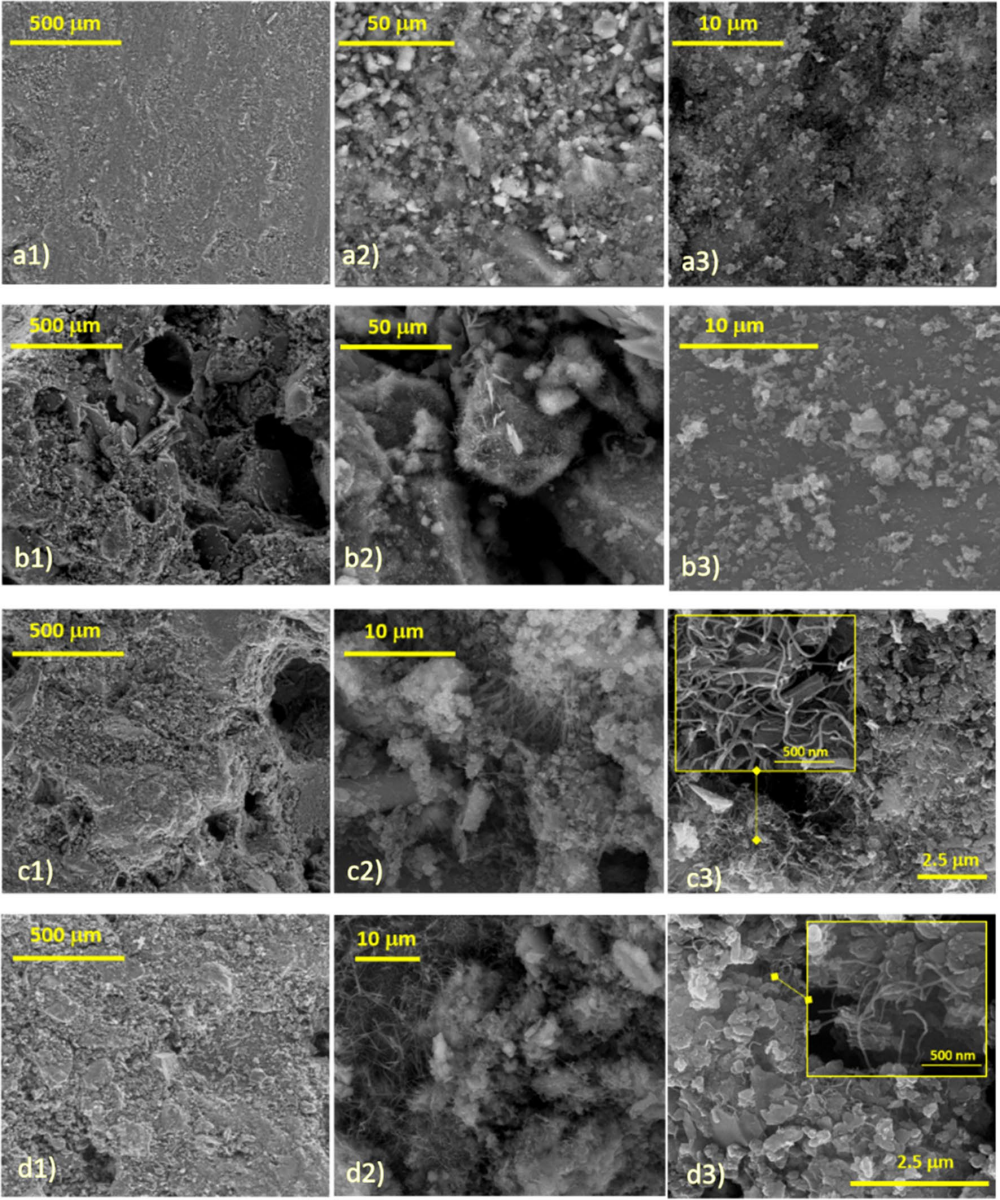
SEM images at different magnifications of (**a**) M_0_, (**b**) M_1_, (**c**) CM_2_, and (**d**) CM_3_.

The morphological structure of mortar samples is shown in Fig. 4. The utilization of the D2 and D3 filler dispersions significantly affected the morphological structure of mortars (Fig. 4c, d respectively) as compared with the M_0_ control sample (Fig. 4a) and M_1_ (produced with D1 dispersion, Fig. 4b). For instance, from SEM micrographs in Fig. 4a1, b1, c1, d1, a2, b2, c2, d2, the presence of a diffuse porosity^43,44^ is observed in the structure of the M_1_ and CM_3_ (which could be ascribed to the presence of NR latex dispersions) and CM_2_ samples (which could be ascribed to a poor interaction between the carbonaceous filler with the cementitious matrix), conversely the M_0_ composite looks more compact. Furthermore, M_0_ and M_1_ highlight the typical amorphous structure of cement-based materials whereas the cement hydrated products (CSH and Hydrated Aluminate phases, CA) were not clearly highlighted (see the SEM pictures in Fig. 4a2, a3)^27,28,45^. As also evidenced by WAXD analysis, the addition of carbonaceous fillers (D2 and D3 aqueous dispersions) promote, speeding-up, the formation of hydrated phases, and the characteristic star-like structure of CSH was observed for CM_2_ (see the SEM microstructure in Fig. 4c2, c3) and CM_3_ (see the SEM microstructure in Fig. 4d2)^27,28^. In addition, for the composite mortars the presence of MWCNTs was clearly detected (see the inset in the Fig. 4c3, d3)^46^. More difficult is to detect the presence of rGO which was dispersed as nanoparticles in the matrix and disappears among the several phases present in the mortars. In particular, MWCNTs present in CM_3_ (Fig. 4d2, d3) are homogenously distributed, mainly as single carbon nanotubes or small bundles and intertwined with the hydrated phases of the cement and some rGO platelets. On the other side, for the CM_2_ sample (Fig. 4c2, c3) the MWCNTs appear mainly as coarse aggregates, evidencing their difficulties in distributing homogeneously in the hydrophilic environment of composite mortars. The results confirm the beneficial effect of the NR latex dispersion to endow a better distribution of the carbonaceous filler throughout the volume of hydrated cementitious phases, avoiding the formation of detrimental coarse aggregates.

### Mechanical and piezoresistive characterization

**Figure 5:**
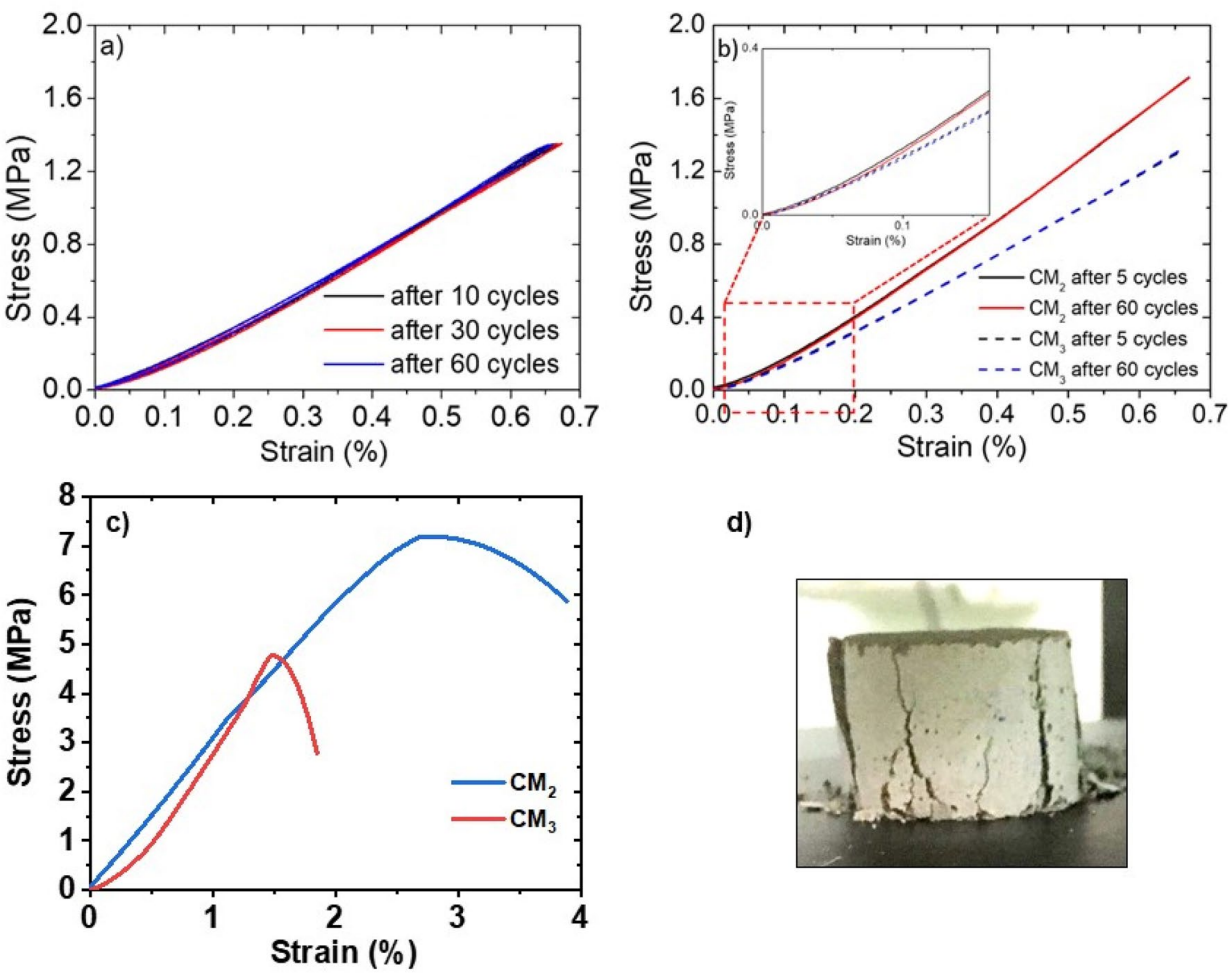
(**a**) Compressive stress–strain curves after 10, 30 and 60 cycles for CM_3_, (**b**) compressive stress–strain curves of CM_2_ and CM_3_ samples after 5 and 60 loading/unloading cycles; (**c**) compressive stress–strain curves of CM_2_ and CM_3_ samples until the breaking point (corresponding to the strain at which the testing machine was not able to control the crack propagation), (**d**) picture of CM_2_ sample at breaking point.

To investigate the piezoresistive behavior of cement-based mortars, the time-dependent electrical resistance (*R*) variations, over 100 mechanical compression cycles for CM_2_ and CM_3_ samples was measured. The samples were submitted to a relatively small compressive deformation (strain < 0.6%) in order to study both the mechanical and piezoresistive properties in the linear elastic region (Fig. 5a).

Compressive elastic modulus values (*E*_C_) of composite samples were calculated from the compressive stress–strain curves reported in Fig. 5b by applying the Eq. 1):

1 $$ \sigma = E_{C} \cdot \varepsilon $$

where the stress (σ, MPa), computed as measure force divide by the cross-section area loaded without any friction reducer, is shown to have a linear dependence on the strain (ε, mm/mm) in the range 0.1–0.3 MPa, with the elastic modulus (*E*c, MPa) as constant of proportionality, also representative of the slope of the linear section. *E*_C_ values of 294 ± 15 MPa and 209 ± 27 MPa were calculated for CM_2_ and CM_3_ systems respectively, after 5 loading/unloading cycles. CM_3_ composite shows a value of the compressive stress (*σ*_C_) at strain of ~ 0.6% lower than that of CM_2_ composite. Hence, the addition of the NR latex in the mortar matrix brings about a decrease of *E*_C_ and *σ*_C_ values, although the filler aqueous dispersions promote a better hydration of cementitious anhydrous phases, as confirmed by WAXD and SEM analysis. Finally, it is worth noting that for the composite mortar CM_2_, the stress–strain curves after 5 and 60 loading/unloading compression cycles slightly differ each other, highlighting a reduction of the mechanical performances as consequence of the compression cycles. This detrimental effect is markedly reduced for the sample CM_3_, highlighting the capability of natural rubber inclusions to improve the deformation of the composite mortar, avoiding the localization of internal microcracks in the elastic deformation region. In fact, the NR affects the mechanical behaviour of mortars, contributing to weakening the interfacial transition zone^46^ between the aggregated and the hydrated phases of cement and promoting the formation of voids (as confirmed by SEM images in Fig. 4). Turki et al.^47^ found a decrease of *E*_C_ and *σ*_C_ values of rubber-based mortars as increasing the rubber content, and ascribed it to the weak interactions of the interfacial transition zone between rubber aggregates and the cementitious phases. Moreover, the decrease of the compressive modulus is also associated to the intrinsic elastic properties of the NR. Indeed, Turatsinze et al. demonstrated that the incorporation of rubber particles in cement paste, if on the one hand reduces the compressive modulus and stress of the cement-based composites, on the other hand, is beneficial in terms of strain and toughness capacity. The rubber material acts as crack arrester and gives rise to cementitious mortars which adsorb more of the compression energy before displaying a macroscopic crack and the consequent structural collapse. Finally, they also pointed out at the beneficial effect of rubber on the reduction of cracking extent from shrinkage, which significantly contributes to the mechanical properties of composite mortars^48^. Similar results were also recently obtained by Gampanart Sukmak et al., who published on the positive effect of the addition of NR latex to improving the flexural strength and toughness of composite mortar through the formation of a rubber-based film which permeate the structure, whilst retarding the setting time and hydration process^49^. Such outcomes were also proved for the composites system presented in this work, when the samples underwent a compression test. As reported in Fig. 5c, the CM_3_ system exhibited an increase of the modulus for strains higher than 0.7%. This is associated with the presence of NR in the inorganic matrix which is responsible for the inclusion of voids in sample manufacturing. When the compression reaches higher strains, the material surrounding the cavities will compact, thus giving a rise to a variation of the sample rigidity over deformation. However, the CM_3_ has a lower maximal stress with respect to CM_2_ (4.76 and 7.19 MPa, respectively), since the latter has a lower presence of voids. Both samples, when the highest stress is reached, present macrocracks (e.g., see in Fig. 5d, the CM_2_ sample at the end of the test).

**Figure 6:**
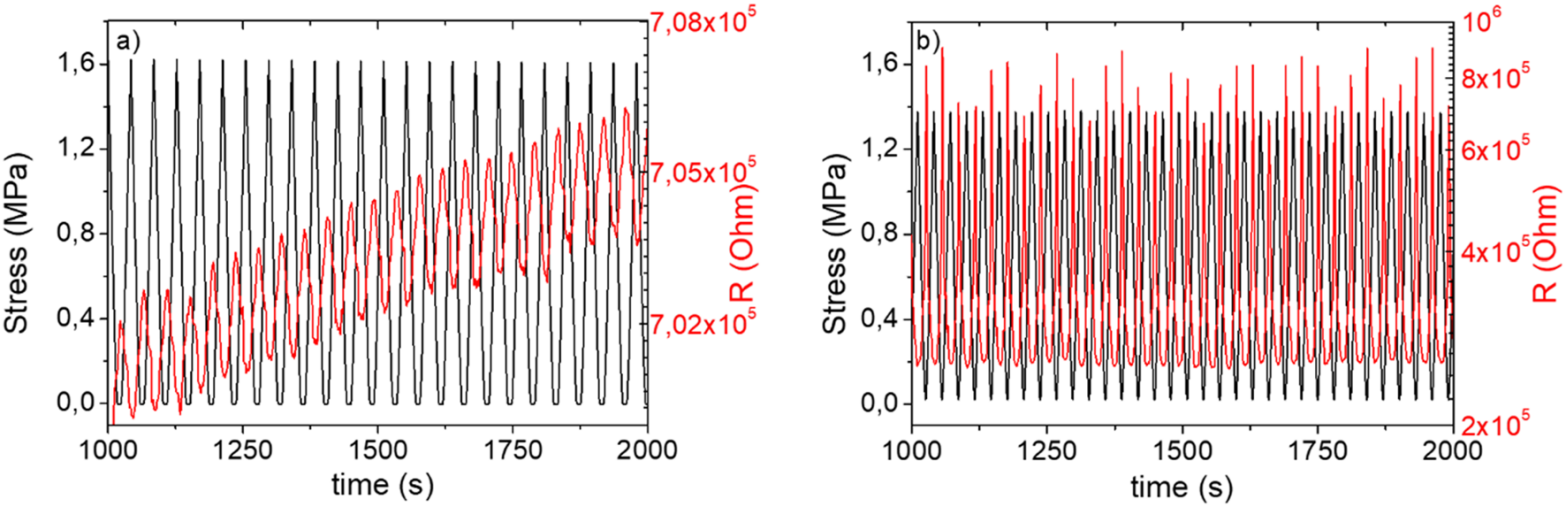
Piezoresistive behaviour, in terms of variation of compression stress and electrical resistance for the systems CM_2_ (**a**) and CM_3_ (**b**).

Piezoresistive results of Fig. 6 show that the CM_2_ mortar (Fig. 6a) does not have a reproducible electrical behavior over loading/unloading cycles; in fact, the electrical resistance increases with the number of loading/unloading cycles. This increase may be ascribed to both an electric polarization of fillers in the cement-based composite^50,51^ and to a modification of the MWCNTs and rGO fillers spatial distribution in the mortar matrix. Indeed, during loading/unloading compressive cycles, local cracks and slips may occur, as also confirmed by the mechanical results (see Fig. 5b). The microcracks modify the conductive percolative network of carbonaceous fillers in the cement-based binding phase and likely, some nanoparticles, i.e. MWCNTs and rGO irreversibly separated or disconnected from each other with a consequent increase of the electrical resistance^52,53^.

On the contrary, the CM_3_ (Fig. 6b) composite, which has a better strain capability due to the presence of rubber phase (i.e., it is able to deform more than the CM_2_ mortar, when the same stress is applied to the sample), exhibits a regular resistance variation over loading and unloading cycles, without any performance degradation. That suggests that the mortar modified with rubber has a relatively high reliability in strain-sensing processes. As shown in Fig. 6b, the electrical resistance decreases with the increasing compressive strain and increases as the compressive strain decreases, changing between the maximum and minimum values at different cycles. This behavior confirms that the carbonaceous filler densifies during compression, realizing more effective contacts which improve the electrical conductivity. When the compression load is released, the carbonaceous fillers recover their initial spatial distribution, and that brings the electrical resistance to its initial value. Therefore, the piezoresistive behavior of the mortars, both CM_2_ and CM_3,_ is connected with the re-construction and de-construction of the conductive network over loading/unloading compressive cycles. Moreover, the variation of the electrical resistance for the CM_3_ composite, after a single compression cycle at ~ 0.6% strain, is higher than that of the CM_2_ composite. These results suggest that the presence of the NR in the CM_3_ sample generates a better spatial arrangement of the carbonaceous filler, contributing to increasing the sensitivity and piezoresistive properties of the composite. Similar conclusions have been already found by Wang et al.^54^ for MWCNTs/polydimethylsiloxane nanocomposites with a segregated structure of the carbon filler.

The sensitivity of piezoresistive material can be evaluated by the gauge factor, GF, defined as the ratio of the relative resistance change (Δ*R*/*R*_0_) to the applied strain. GF values of 0.34 and of 0.70 have been found for CM_2_ and CM_3_ respectively. The higher piezoresistive sensitivity of the CM_3_ sample is ascribed to a more efficient de-construction and re-construction of the fillers network during compression cycles.

**Figure 7:**
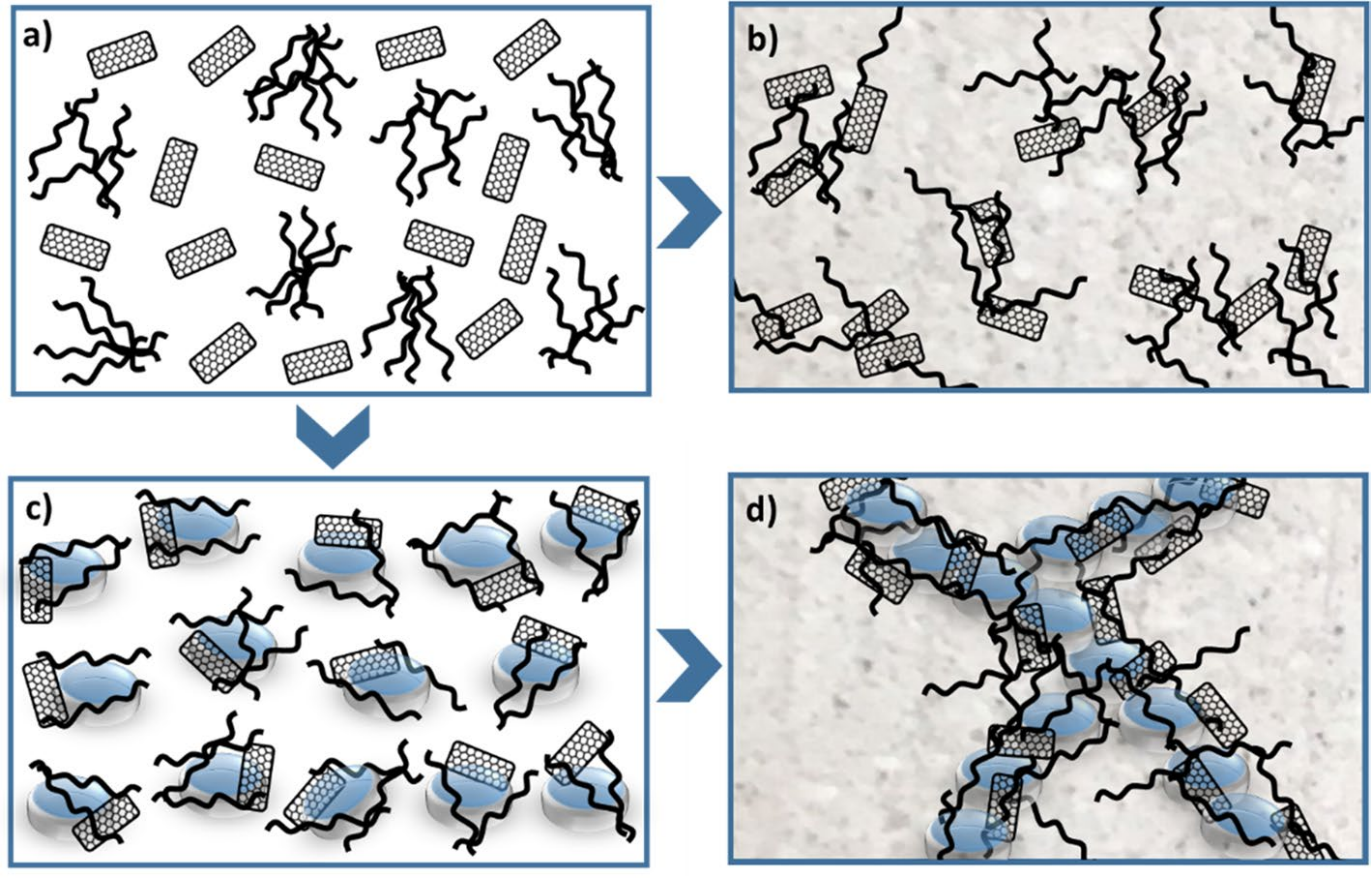
Schematic representation of the rGO/MWCNTs fillers spatial distribution in the mortars induced by presence/absence of natural rubber. Filler dispersion obtained without NR (**a**) and with self-assembling on NR latex particles (**b**), random filler distribution within the mortar matrix without NR (**c**) and percolated filler network assisted by NR latex particles segregation (**d**).

The different piezoresistive behavior of the mortars, CM_2_ and CM_3_ can be rationalized by taking into account the different spatial distributions of the carbonaceous filler in the two samples, which occur during their preparation process. In fact, when rGO and MWCNTs fillers are dispersed in absence of NR (Fig. 7a) and then added in the mortar matrix, they are distributed all over the entire volume with a random filler distribution (as schematically reported in Fig. 7b) and with a tendency to form aggregates, as revealed by SEM analysis of Fig. 4. In this spatial distribution the filler contact probability is low and thus also the possibility to realize a conductive percolative network. In the other case, when rGO/MWCNTs assembled onto the surface of NR particles (Fig. 7c) are added in the cementitious phase, they form a three-dimensional filler network in which rGO and MWCNTs are localized only in between the interstices of the single latex particles which coalesce to produce a continuous rubbery phase within the cementitious matrix (Fig. 7d). This spatial distribution of the fillers, which are confined in a smaller volume than that without NR, increases the contact points among fillers on the submicron scale, which is important to realize electrical conductive paths and, therefore, increases the electrical and piezoresistive properties of the CM_3_ sample.

Finally the presence of NR coupled with the carbonaceous fillers plays a double effect in the composite mortars: (1) it contributes to reducing both the compressive modulus of the sample CM_3_ compared to that of the sample CM_2_ and the occurring of local cracks and slips during repeated compression cycles, thus avoiding irreversible modifications in the spatial arrangement of fillers^48^; (2) it contributes to building-up a more effective conductive and segregated filler network within the mortar matrix, which facilitates the re-construction and de-construction of conductive percolation paths as compared with the randomly filler structure obtained in the CM_2_ system without NR.

The effect of segregated carbon filler structures in enhancing piezoresistive properties of polymer and natural rubber-based composites has already been investigated^55,56^, while, as far as we know, it has never been highlighted in cement-based composites.

Hence, the presence of the NR latex, inducing better segregated structures of the rGO/MWCNTs fillers increase the sensitivity and electrical conductivity of composite mortars, indicating that the utilization of rubber-based carbonaceous filler dispersions as raw material for the preparation of mortars is a valuable method in developing effective mortar-based piezoelectric sensors, which can be potentially used for developing smart buildings and infrastructures.

## Conclusions

Cement-based composites with a rGO/MWCNTs well-distributed morphology were prepared by using an innovative approach consisting of assembling carbonaceous fillers onto NR latex particles with subsequent addition in the cement matrix. The effects of natural-rubber latex on distribution of carbonaceous fillers into the cementitious phase and on the resulting structural, morphological mechanical and piezoresistive properties of composite mortars were thoroughly investigated. The Inelastic Neutron Scattering results highlight that the average structure and dynamics of hydrogen within the mortars are not affected by the inclusion of natural rubber, rGO, and MWCNTs at the investigated concentrations. Moreover, negligible molecular interactions take place between NR with the rest of components of the mortar matrix which do not affect the hydration processes of cement particles. On the other side, X Ray diffraction and morphological analysis show that the carbonaceous fillers speed-up the formation of hydrated phases (Hydrated Calcium Silicate, CSH) enhancing the assembly of the calcium hydroxide (CH) crystals produced during the hydration process. Furthermore, SEM analysis reveals that the NR latex leads to a more homogeneous and percolated distribution of carbonaceous fillers throughout the volume of hydrated cementitious phases, as compared to the composite mortar obtained without NR.

Finally, the inclusion of natural rubber contributes to improve the deformation capability of the composite mortar and induces a more homogeneous filler distribution into the mortar matrix which increases the piezo-resistive sensitivity of composites compared to the system produced without rubber, which evidences the presence of MWCNTs/rGO coarse aggregates. Therefore, the obtained results suggest that the utilization of rubber-based conductive filler composite dispersion as a raw material for the preparation of composite mortars is a valuable method in developing cement-based effective and reliable piezoresistive strain sensors.

## Materials

Portland cement (Type IV, Mapei Spa, Milan, Italy)^57,58^ and normalized sand (CEN-Standard Sand according to EN 196-1, grain size distribution ranging in 0.08–2.00 mm, Mapei Spa, Milan, Italy) were used in the preparation of cement-based samples. Tap water, conforming to European Standard EN 1008:2004^59^ was used to prepare mortar specimens.

Prevulcanized natural rubber latex (NR) (HMR 10, solid content: 60.5 wt%) was supplied by Synthomer, UK. Reduced Graphene Oxide was produced through the chemical reduction of Graphene Oxide by ascorbic acid (AA) treatment, which was supplied by VWR Chemicals. Multi Walled Carbon Nanotubes (MWCNT) (NC 7000, diameter: 10 nm, length: 1.5 μm, density: 1.75 g/cm^3^) were purchased from Nanocyl S.A., Belgium. Cetyltrimethylammonium bromide (CTAB), as a surfactant, was obtained from Sigma Chemicals Company.

## Sample preparation

### Preparation of reduced graphene oxide (rGO)

rGO was prepared through the chemical reduction of GO assisted by ascorbic acid, accordingly to a consolidated procedure^60^. In details, GO (1.65 mg/mL), obtained by graphite through a modified Hummers method^61^, was dispersed in water by using an ultrasonic bath (temperature: 25 °C, frequency: 40 kHz, amplitude: 100%) for 30 min. Ascorbic acid was added to the GO dispersion and the mixture was stirred at 60 °C for 4 h. The GO/AA weight ratio was fixed to 1:20. The obtained rGO dispersion was filtered, and the particles washed with distilled water to remove the excess of ascorbic acid.

### Preparation of filler-based aqueous dispersions

rGO, MWCNTs and CTAB (with a weight ratio rGO/MWCNTs/CTAB equal to 1:1:2) were dispersed into tap water by using an ultrasound probe (temperature: 0 °C, cycle: 0.5, amplitude: 80%) for 30 min. NR latex was added into the aforementioned rGO/MWCNT/CTAB dispersion and, further, sonicated for 30 min in order to obtain a stable fillers/NR latex dispersion (more details of fillers/NR latex preparation is well described in Zhan et al.^16^). In order to investigate the roles of carbonaceous fillers (mixture 1:1 wt/wt of rGO and MWCNTs) and NR on hydration mechanisms of the anhydrous cementitious phases, control composite samples containing only fillers and only the NR latex particles were also prepared. Therefore, for the realization of composite mortars three different filler-based aqueous dispersions were prepared (see Table 1).

**Table 1:** Formulation of filler-based aqueous dispersions.

Filler dispersion	H_2_O (wt%)	NR-latex (wt%)	MWCNT (wt%)	rGO (wt%)	CTAB (wt%)
D1	84.40	15.60	–	–	–
D2	97.76	–	0.56	0.56	1.12
D3	89.18	8.90	0.48	0.48	0.96

### Preparation of cement-based mortars

The cement-based mortars were manufactured first by mixing and dry-homogenizing suitable amount of the powders (cement and standard sand). Subsequently, an appropriate amount of filler-based aqueous dispersions (D1, D2 and D3) was gradually added to the dry mixture in order to be uniformly mixed (the details of the mix-design formulations are reported in Table 2). The wet mixture was then poured in a 2 × 2 × 2 cm^3^ molds, and the whole system was cured in a climatic chamber (20 °C and 100% RH). After 28 days of hydration curing, the samples were demolded and put in an oven at 40 °C for 24 h. A reference mortar produced by using only water was also prepared (M_0_). All the composites were then comprehensively characterized in terms of chemical, morphological and functional properties (i.e. piezoresistive properties).

**Table 2:** Formulations of the manufactured composite mortars.

Sample	Cement (wt%)	Sand (wt%)	H_2_O (wt %)		D1 (wt%)	D2 (wt%)	D3 (wt%)
M_0_	22	66	12	–		–	–
M_1_	22	66	–	12		–	–
CM_2_	22	66	–	–		12	–
CM_3_	22	66	–	–		–	12

## Methods

To assess the effect of the several filler-based dispersions (including the dispersion made of pristine rubber latex) on cement hydration process, the produced cement-based composites were characterized by means of Inelastic Neutron Scattering (INS)^23,24,26^. In details, INS experiments were performed on the TOSCA spectrometer^24,62^ at the ISIS Neutron and Muon Source, UK. Samples were loaded in flat Al containers and measured within a closed-cycle refrigerator at the temperature of 20 K, to minimize the Debye–Waller factor^25^. The several filler dispersions (described in Table 1) were loaded in 1-mm-thick indium-sealed containers, while solid samples (mortars) were placed as prepared, without the need to grind the samples, with approximate thickness of 2.5 mm. Aluminum is generally used as sample container owing to its low scattering cross section for neutrons^62^, thus providing a negligible self-attenuation correction^63^.

Mineralogical phases evolution during hydration curing of cement phases were evaluated by means of XRD using a Panalytical X'Pert Pro diffractometer equipped with PixCel 1D detector (operative conditions: CuKα1/Kα2 radiation, 40 kV, 40 mA, 2Θ range from 5° to 80°, step size 0.0131° 2Θ, counting time 40 s per step). In this case, the cement-based samples were finally milled before the XRD analysis.

The microstructural and morphological structure of new phases originated from cement hydration and their interactions with the carbonaceous and rubber-based filler were evaluated by Scanning Electron Microscopy, SEM, (FEI Quanta 200 FEG scanning electron microscope-ESEM, Eindhoven, The Netherlands). The mortar samples were frozen in liquid nitrogen and quickly broken, gold sputtered, and analyzed at an accelerating voltage of 20 kV.

The experimental setup for the evaluation of the mechanical and piezoresistive properties was realized by using a mechanical tester (Instron 5564 dynamometer) and a multimeter (Agilent 34401A 6½ Digit Multimeter) controlled by a homemade LabVIEW program. The multimeter was connected with two electrodes (adopting the 2-probe measurement method) to the composite samples (cubic samples of 1.5 cm^3^), and the change in the electrical resistance of the specimen submitted out to loading and unloading cycles was continuously monitored through a dedicated computer. A preload of 200 N was applied to ensure an effective contact of the electrodes along the entire contact surface (copper conductive tape was glued the sample surfaces of the cubic sample). The electrical resistance changes were evaluated by submitting the composite samples at 100 cyclic loading/unloading, with 0.5% deformation and 0.3 mm/min actuation rate, at room temperature.

## References

[1] Dimov D (2018). Ultrahigh performance nanoengineered graphene-concrete composites for multifunctional applications. Adv. Funct. Mater..

[2] Chuah S, Pan Z, Sanjayan JG, Wang CM, Duan WH (2014). Nano reinforced cement and concrete composites and new perspective from graphene oxide. Constr. Build. Mater..

[3] Sun S (2017). Nano graphite platelets-enabled piezoresistive cementitious composites for structural health monitoring. Constr. Build. Mater..

[4] Reddy PN, Kavyateja BV, Jindal BB (2020). Structural health monitoring methods, dispersion of fibers, micro and macro structural properties, sensing, and mechanical properties of self-sensing concrete-A review. Struct. Concr..

[5] Hongyu S, Binmeng C, Bo L, Shengwen T, Zongjin L (2017). Influence of dispersants on the properties of CNTs reinforced cement-based materials. Constr. Build. Mater..

[6] Zhang L (2018). Effect of characteristics of assembly unit of CNT/NCB composite fillers on properties of smart cement-based materials. Compos. Part A Appl. Sci. Manuf..

[7] Zhan M, Pan G, Zhou F, Mi R, Shah SP (2020). In situ-grown carbon nanotubes enhanced cement-based materials with multifunctionality. Cem. Concr. Compos..

[8] Han B (2015). Electrostatic self-assembled carbon nanotube/nano carbon black composite fillers reinforced cement-based materials with multifunctionality. Compos. Part A Appl. Sci. Manuf..

[9] Konsta-Gdoutos MS, Danoglidis PA, Falara MG, Nitodas SE (2017). Fresh and mechanical properties, and strain sensing of nanomodified cement mortars: The effects of MWCNT aspect ratio, density and functionalization. Cem. Concr. Compos..

[10] Han J, Pan J, Cai J, Li X (2020). A review on carbon-based self-sensing cementitious composites. Constr. Build. Mater..

[11] Parveen S, Rana S, Fangueiro R (2013). A review on nanomaterial dispersion, microstructure, and mechanical properties of carbon nanotube and nanofiber reinforced cementitious composites. J. Nanomater..

[12] Ur Rehman SK, Kumarova S, Memon SA, Javed MF, Jameel M (2020). A review of microscale, rheological, mechanical, thermoelectrical and piezoresistive properties of graphene based cement composite. Nanomaterials.

[13] Hossain MM, Karim R, Hasan M, Hossain MK, Zain MFM (2016). Durability of mortar and concrete made up of pozzolans as a partial replacement of cement: A review. Constr. Build. Mater..

[14] Xu Y (2018). A holistic review of cement composites reinforced with graphene oxide, review. Constr. Build. Mater..

[15] Bai S, Jiang L, Xu N, Jin M, Jiang S (2018). Enhancement of mechanical and electrical properties of graphene/cement composite due to improved dispersion of graphene by addition of silica fume. Constr. Build. Mater..

[16] Zhan Y (2020). An anisotropic layer-by-layer carbon nanotube/boron nitride/rubber composite and its application in electromagnetic shielding. Nanoscale.

[17] Boratto MH, Nozella NL, Ramos RA, Aparecido da Silva R, Graeff CFO (2020). Flexible conductive blend of natural rubber latex with PEDOT: PSS. APL Mater..

[18] Guerra NB (2021). Biomedical applications of natural rubber latex from the rubber tree *Hevea brasiliensis*. Mater. Sci. Eng. C.

[19] Zhan Y, Lavorgna M, Buonocore GG, Xia H (2012). Enhancing electrical conductivity of rubber composites by constructing interconnected network of self-assembled graphene with latex mixing. J. Mater. Chem..

[20] Salzano De Luna M (2019). Nanocomposite polymeric materials with 3D graphene-based architectures: From design strategies to tailored properties and potential applications. Prog. Polym. Sci..

[21] Toghroli A (2018). A review on pavement porous concrete using recycled waste materials. Smart Struct. Syst..

[22] Letelier V, Tarela E, Muñoz P, Moriconi G (2016). Assessment of the mechanical properties of a concrete made by reusing both: Brewery spent diatomite and recycled aggregates. Constr. Build. Mater..

[23] Parker SF (1997). TOSCA: A world class inelastic neutron spectrometer. Phys. B Condens. Matter.

[24] Fernandez-Alonso F, Price DL (2013). Neutron scattering—fundamentals.

[25] Mitchell PCH (2005). Vibrational spectroscopy with neutrons: with applications in chemistry, biology, materials science and catalysis.

[26] Ricci MA, Nardone M, Fontana A, Andreani C, Hahn W (1998). Light and neutron scattering studies of the OH stretching band in liquid and supercritical water. J. Chem. Phys..

[27] Senesi R (2013). The quantum nature of the OH stretching mode in ice and water probed by neutron scattering experiments. J. Chem. Phys..

[28] Senesi R, Romanelli G, Adams MA, Andreani C (2013). Temperature dependence of the zero point kinetic energy in ice and water above room temperature. Chem. Phys..

[29] Squires GL (1996). Introduction to the theory of thermal neutron scattering.

[30] Festa G (2019). Old burned bones tell us about past cultures. Spec. Eur..

[31] Thomas JJ, Chen JJ, Jennings HM, Neumann DA (2003). Ca–OH bonding in the C–S–H gel phase of tricalcium silicate and white Portland cement pastes measured by inelastic neutron scattering. Chem. Mater..

[32] Bordallo HN, Aldridge LP, Desmedt A (2006). Water dynamics in hardened ordinary portland cement paste or concrete: From quasielastic neutron scattering. J. Phys. Chem..

[33] Peterson VK, Brown CM, Livingston RA (2006). Quasielastic and inelastic neutron scattering study of the hydration of monoclinic and triclinic tricalcium silicate. Chem. Phys..

[34] Faraone A, Fratini E, Baglioni P, Chen SH (2004). Quasielastic and inelastic neutron scattering on hydrated calcium silicate pastes. J. Chem. Phys..

[35] Pellenq RJ-M (2009). A realistic molecular model of cement hydrates. Proc. Natl. Acad. Sci..

[36] Yu P, Kirkpatrick RJ, Poe B, McMillan PF, Cong X (1999). Structure of calcium silicate hydrate (C–S–H): near-, mid-, and far-infrared spectroscopy. J. Am. Ceram. Soc..

[37] Adams MA, Gabrys BJ, Zajac WM, Peiffer DG (2005). High-resolution incoherent inelastic neutron scattering spectra of polyisobutylene and polyisoprene. Macromolecules.

[38] ISIS INS Database, 2020. https://www.isis.stfc.ac.uk/Pages/INS-database.aspx.

[39] Omotoso OE, Ivey DG, Mikula R (1998). Hexavalent chromium in tricalcium silicate Part I quantitative X-ray diffraction analysis of crystalline hydration products. J. Mater. Sci..

[40] Xu G, Du S, He J, Shi X (2019). The role of admixed graphene oxide in a cement hydration system. Carbon.

[41] Lin C, Wei W, Hu YH (2016). Catalytic behavior of graphene oxide for cement hydration process. J. Phys. Chem. Solids.

[42] Lia W (2017). Effects of graphene oxide on early-age hydration and electrical resistivity of Portland cement paste. Constr. Build. Mater..

[43] De Luca Bossa F (2020). Greener nanocomposite polyurethane foam based on sustainable polyol and natural fillers: investigation of chemico-physical and mechanical properties. Materials.

[44] Stanzione M (2020). Tuning of polyurethane foam mechanical and thermal properties using ball-milled cellulose. Carbohydr. Polym..

[45] Tambara Júnior LUD, Cheriaf M, Rocha JC (2018). Development of alkaline-activated self-leveling hybrid mortar ash-based composites. Materials.

[46] Prokopski G, Halbiniak J (2000). Interfacial transition zone in cementitious materials. Cem. Concr. Res..

[47] Turki M, Bretagne E, Rouis MJ, Quéneudec M (2009). Microstructure, physical and mechanical properties of mortar–rubber aggregates mixtures. Constr. Build. Mater..

[48] Turatsinze A, Bonnet S, Granju J-L (2005). Mechanical characterisation of cement-based mortar incorporating rubber aggregates from recycled worn tyres. Build. Environ..

[49] Sukmak G (2020). Physical and mechanical properties of natural rubber modified cement paste. Constr. Build. Mater..

[50] Rao R, Sindu BS, Sasmal S (2020). Synthesis, design and piezo-resistive characteristics of cementitious smart nanocomposites with different types of functionalized MWCNTs under long cyclic loading. Cem. Concr. Compos..

[51] Wen S, Chung DDL (2007). Partial replacement of carbon fiber by carbon black in multifunctional cement–matrix composites. Carbon.

[52] Tao J, Wang X, Wang Z, Zeng Q (2019). Graphene nanoplatelets as an effective additive to tune the microstructures and piezoresistive properties of cement-based composites. Constr. Build. Mater..

[53] Yoo DY, You I, Lee SJ (2018). Electrical and piezoresistive sensing capacities of cement paste with multi-walled carbon nanotubes. Arch. Civ. Mech. Eng..

[54] Wang M (2017). Enhanced electrical conductivity and piezoresistive sensing in multi-wall carbon nanotubes/polydimethylsiloxane nanocomposites via the construction of a self-segregated structure. Nanoscale.

[55] Zhai W (2018). Segregated conductive polymer composite with synergistically electrical and mechanical properties. Compos. Part A Appl. Sci. Manuf..

[56] Lin Y (2016). Graphene−elastomer composites with segregated nanostructured network for liquid and strain sensing application. ACS Appl. Mater. Interfaces.

[57] Verdolotti L, Di Maio E, Lavorgna M, Iannace S (2012). Hydration-induced reinforcement of rigid polyurethane–cement foams: Mechanical and functional properties. J. Mater. Sci..

[58] Verdolotti L, Di Maio E, Forte G, Lavorgna M, Iannace S (2010). Hydration-induced reinforcement of polyurethane-cement foams: Solvent resistance and mechanical properties. J. Mater. Sci..

[59] Mixing Water for Concrete-Specification for Sampling, Testing and Assessing the Suitability of Water, Including Water Recovered from Processes in the Concrete Industry, as Mixing Water for Concrete; BSI: London, UK, **10**, 1008 (2002).

[60] Fernandez-Merino MJ (2010). Vitamin C is an ideal substitute for hydrazine in the reduction of graphene oxide suspensions. J. Phys. Chem. C.

[61] Hummers WS, Offeman RE (1958). Preparation of graphitic oxide. J. Am. Chem. Soc..

[62] Rudic, S., *et al*. TOSCA international beamline review. STFC Technical Report (2013).

[63] Sears VF (1992). Neutron scattering lengths and cross sections. Neutron News.

[64] Scatigno C (2020). A python algorithm to analyze inelastic neutron scattering spectra based on the y-scale formalism. J. Chem. Theor. Comput..

